# Isolation and Total Synthesis of Stolonines A–C, Unique Taurine Amides from the Australian Marine Tunicate *Cnemidocarpa stolonifera*

**DOI:** 10.3390/md13074556

**Published:** 2015-07-22

**Authors:** Trong D. Tran, Ngoc B. Pham, Merrick Ekins, John N. A. Hooper, Ronald J. Quinn

**Affiliations:** 1 Eskitis Institute for Drug Discovery, Griffith University, Brisbane, Queensland 4111, Australia; E-Mails: trong.tran@griffithuni.edu.au (T.D.T.); n.pham@griffith.edu.au (N.B.P.); john.hooper@qm.qld.gov.au (J.N.A.H.); 2 Queensland Museum, Brisbane, Queensland 4101, Australia; E-Mail: merrick.ekins@qm.qld.gov.au

**Keywords:** *Cnemidocarpa stolonifera*, taurine amide, PC3 cell line, immunofluorescence assay

## Abstract

*Cnemidocarpa stolonifera* is an underexplored marine tunicate that only occurs on the tropical to subtropical East Coast of Australia, with only two pyridoacridine compounds reported previously. Qualitative analysis of the lead-like enhanced fractions of *C. stolonifera* by LC-MS dual electrospray ionization coupled with PDA and ELSD detectors led to the identification of three new natural products, stolonines A–C (**1**–**3**), belonging to the taurine amide structure class. Structures of the new compounds were determined by NMR and MS analyses and later verified by total synthesis. This is the first time that the conjugates of taurine with 3-indoleglyoxylic acid, quinoline-2-carboxylic acid and β-carboline-3-carboxylic acid present in stolonines A–C (**1**–**3**), respectively, have been reported. An immunofluorescence assay on PC3 cells indicated that compounds **1** and **3** increased cell size, induced mitochondrial texture elongation, and *caused apoptosis in PC3 cells*.

## 1. Introduction

The exploration of marine secondary metabolites only began in the early 1950s [1] with the landmark identification of the two nucleosides spongothymidine and spongouridine from the Caribbean marine sponge *Cryptotethia crypta* [2,3]. Since then, marine natural product discovery has increased annually and has accelerated over the last two decades [4]. By 2010 more than 15,000 new marine natural products were discovered with 8368 new compounds recorded in a decade between 2001 and 2010 [5]. Approximately 75% of marine natural products were isolated from marine invertebrates [4] from which sponges, tunicates, bryozoans or molluscs have provided the majority of the compounds involved in clinical or preclinical trials [5]. It is expected that the discovery of marine natural products will provide new and improved therapeutics for human illnesses, along with other innovative products for other industrial activities (e.g., nutraceutics and biotechnology) [6].

To maximize the chance to discover new marine natural products, a library of fractions from rare or restricted marine invertebrates was generated from the Nature Bank database [7]. Qualitative analysis of lead-like enhanced fractions of samples in the selected library by LC-MS dual electrospray ionization coupled with PDA and ELSD detectors [8,9,10] indicated that the marine tunicate *Cnemidocarpa stolonifera* contained two different compound classes in two different fractions. The first class showed strong MS signals in a positive mode and strong UV absorptions at 380 nm while the second one displayed strong MS signals in a negative mode and strong UV absorptions at 280 nm. Mass-guided isolation of the marine tunicate *C. stolonifera* extract led to the isolation of two known pyridoacridine alkaloids, 11-hydroxyascididemin (**4**) and cnemidine A (**5**) [11], accounting for the first compound class. For the second class of compounds, three new taurine amide derivatives, stolonines A–C (**1**–**3**), were isolated.

Taurine is found at high concentration in mammalian plasma and tissues [12]. This compound has been known to exhibit a large spectrum of physiological functions in the liver, kidney, heart, pancreas, retina, and brain [13,14]. Its depletion is associated with several disease conditions such as diabetes, Parkinson’s, Alzheimer’s, cardiovascular diseases, and neuronal damages in the retina [13]. So far, 81 taurine derivatives from natural sources have been reported of which 47 compounds are amides produced between taurine with fatty acids, steroid acids or other aromatic acid residues [15]. The occurrence of the conjugates of taurine with 3-indoleglyoxylic acid, quinoline-2-carboxylic acid and β-carboline-3-carboxylic acid present in stolonines A–C (**1**–**3**), respectively, has not been previously reported. This paper describes the isolation, structure elucidation and total synthesis of stolonines A–C (**1**–**3**) from the marine tunicate *C. stolonifera*. Cytotoxicity of **1**–**3** against human prostate cancer PC3 cell line was evaluated and the result indicated that they were not active up to 20 μM. However, an immunofluorescence assay on PC3 cells revealed that compounds **1** and **3** increased cell size, induced mitochondrial texture elongation and caused apoptosis in PC3 cells.

## 2. Results and Discussion

**Figure 1:**
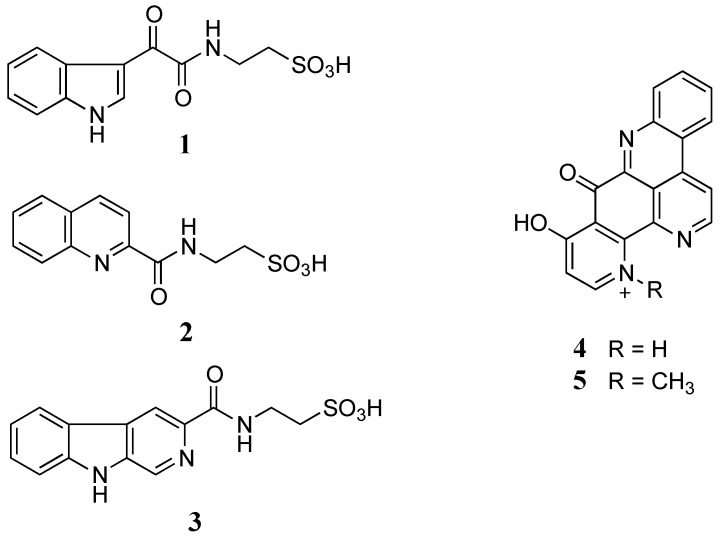
Structures of stolonines A-C (**1**–**3**), 11-hydroxyascididemin (**4**) and cnemidine A (**5**) isolated from the tunicate *C. stolonifera*.

The freeze-dried *C. stolonifera* tunicate was sequentially extracted with *n*-hexane, dichloromethane (DCM), and methanol (MeOH). The DCM/MeOH extracts were then combined and chromatographed using C_18_ bonded silica HPLC MeOH/H_2_O/0.1% trifluoroacetic acid (TFA) to yield three new alkaloids, stolonines A–C (**1**–**3**) together with two known alkaloids, 11-hydroxyascididemin (**4**) and cnemidine A (**5**) (Figure 1).

**Figure 2:**
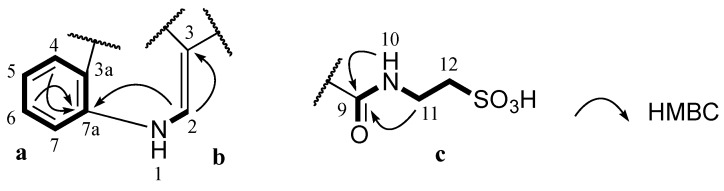
Partial structures (**a**, **b** and **c**) of **1** and their key HMBC correlations.

Stolonine A (**1**) was obtained as a white amorphous solid. The (−)-HRESIMS spectrum displayed a molecular ion [M−H]^−^ at *m*/*z* 295.0390, which was consistent with the molecular formula C_12_H_12_N_2_O_5_S. The IR spectrum indicated the presence of an S=O stretching band at 1205 cm^−1^ [16]. A ^1^H NMR spectrum of **1** showed two exchangeable protons (δ_H_ 12.20 and 8.78 ppm), five aromatic protons (δ_H_ 8.82, 8.22, 7.52, 7.26 and 7.24 ppm) and two methylenes (δ_H_ 3.50 and 2.66 ppm). Further analysis of the ^13^C NMR and gHSQCAD spectra indicated that the molecule contained two carbonyls (δ_C_ 181.6 and 162.7 ppm), eight aromatic carbons (δ_C_ 138.5, 136.2, 126.2, 123.3, 122.4, 121.3, 112.5 and 112.1 ppm) and two methylenes (δ_C_ 49.8 and 35.5 ppm) (Table 1). *J* coupling constants of aromatic protons H-4 (δ_H_ 8.22, dd, 1.8, 7.2 Hz), H-5 (δ_H_ 7.24, dt, 1.8, 7.2 Hz), H-6 (δ_H_ 7.26, dt, 1.8, 7.2 Hz) and H-7 (δ_H_ 7.52, dd, 1.8, 7.2 Hz) and their COSY correlations were characteristic of a 1,2-disubstituted benzene ring (**a**, Figure 2). A gCOSY spectrum displayed correlations from the exchangeable proton H-1 (δ_H_ 12.20, br.s) to H-2 (δ_H_ 8.82, d, 3.0 Hz) and also from H-11 (δ_H_ 3.50, dd, 6.0, 6.6 Hz) to the triplet exchangeable proton H-10 (δ_H_ 8.78, t, 5.4 Hz) and H-12 (δ_H_ 2.66, t, 6.6 Hz) facilitating the establishment of two other spin systems, –NH–CH= (**b**, Figure 2) and –NH–CH2–CH2– (**c**, Figure 2) respectively. The aromatic carbon C-3 (δ_C_ 112.1 ppm) was attached to C-2 (δ_C_ 138.5 ppm) in the moiety **b** determined by a HMBC correlation from H-2 to C-3. A HMBC correlation from H-2 to C-7a (δ_C_ 136.2 ppm) supported the linkage from **a** to **b** at C-7a (Figure 2). Both protons H-10 and H-11 in the moiety **c** showed HMBC correlations with a carbonyl carbon at δ_C_ 162.7 ppm suggesting the connection of this carbonyl to N-10 to form an amide bond (**c**, Figure 2). Two methylene signals at δ_H_ 3.50 and 2.66 ppm corresponding to carbons C-11 (δ_C_ 35.5 ppm) and C-12 (δ_C_ 49.8 ppm) as well as the relatively downfield resonance of the methylene C-12 were diagnostic of methylenes in a taurine moiety (**c**, Figure 2) [17,18,19].

**Table 1:** NMR data for **1** in DMSO-*d_6_* ^a^.

Position	δ_C_	δ_H_ (*J* in Hz)	gCOSY	gHMBCAD
1		12.20, br.s	2	
2	138.5	8.82, d (3.0)	1	3, 3a, 7a
3	112.1			
3a	126.2			
4	121.3	8.22, dd (1.8, 7.2)	5	6, 7a
5	122.4	7.24, dt (1.8, 7.2)	4, 6	3a, 7
6	123.3	7.26, dt (1.8, 7.2)	5, 7	4, 7a
7	112.5	7.52, dd (1.8, 7.2)	6	3a, 5
7a	136.2			
8	181.6			
9	162.7			
10		8.78, t (5.4)	11	9
11	35.5	3.50, dd (6.0, 6.6)	10, 12	9, 12
12	49.8	2.66, t (6.6)	11	11

^a^ ^1^H NMR at 600 MHz referenced to residual DMSO solvent (δ_H_ 2.50 ppm) and ^13^C NMR at 150 MHz referenced to residual DMSO solvent (δ_C_ 39.52 ppm).

**Figure 3:**
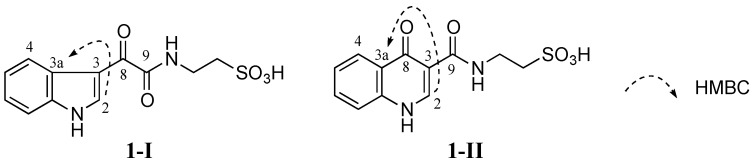
Two possible structures **1-I** and **1-II** of **1** (**1-I** and **1-II** are possible structural isomers of **1**).

No HMBC correlation from any proton to the carbonyl C-8 (δ_C_ 181.6 ppm) was observed when HMBC experiments were performed and optimized with different *J*_HC_ couplings. Therefore, two different isomers **1-I** and **1-II** were conceivable from these data (Figure 3). Detailed HMBC analysis showed that H-2 had a HMBC correlation with C-3a (δ_C_ 126.2 ppm). This suggested that **1** was favorable to **1-I** since the H-2 to C-3a correlation in **1-I** was a three-bond coupling while the H-2 to C-3a correlation in **1-II** was a four-bond coupling (Figure 3).

Density functional theory (DFT) NMR calculations together with the DP4 probability analysis, which have recently emerged as powerful tools for the determination of structures that lack sufficient NMR characterization or contain unusual constituents or connectivities [20,21,22,23,24,25], were employed to verify the assigned structure for **1**. Theoretical ^1^H and ^13^C chemical shifts were calculated at the mPW1PW91/6-31G(d)//B3lyp/6-31G(d) levels. Calculated NMR data of **1-I** and **1-II** were then compared with the experimental NMR data based on the corrected mean absolute error (CMAE) and the DP4 probability (Computational Details, Experimental Section) to determine which of the isomers fit with the experimental data. The results (Table 2) indicated that both ^13^C and ^1^H chemical shifts of the isomer **1-I** showed lower CMAE compared to those of the isomer **1-II**. Significant differences were observed in DP4 probabilities. In particular, **1-I** had 100% probability for ^13^C chemical shifts and 85.3% probability for ^1^H chemical shifts while **1-II** only occupied 0% for ^13^C chemical shifts and 14.7% for ^1^H chemical shifts (Table 2). These probabilities indicated that the assignment of the isomer **1-I** for **1** was at a high level of confidence [20]. Therefore, the structure of **1** was suggested as the isomer **1-I**.

**Table 2:** Comparison of experimental and calculated ^13^C and ^1^H chemical shifts for **1** in DMSO-*d_6_*.

Position	δ_C_ (Exp.)	1-I		1-II		δ_H_ (Exp.)	1-I		1-II	
		δ_C_ (Calc.)	δ_C_ (Scaled)	δ_C_ (Calc.)	δ_C_ (Scaled)		δ_H_ (Calc.)	δ_H_ (Scaled)	δ_H_ (Calc.)	δ_H_ (Scaled)
2	138.5	136.9	140.3	141.3	142.8	8.82	8.74	8.61	8.84	8.67
3	112.1	111.5	112.9	111.2	110.2					
3a	126.2	124.1	126.5	123.0	122.9					
4	121.3	118.9	120.9	124.3	124.4	8.22	8.62	8.48	8.40	8.19
5	122.4	120.1	122.1	121.1	120.9	7.24	7.63	7.40	7.63	7.35
6	123.3	120.9	123.0	129.4	129.9	7.26	7.59	7.36	8.00	7.76
7	112.5	108.7	109.9	114.8	114.1	7.52	7.55	7.31	7.53	7.24
7a	136.2	131.9	134.9	134.4	135.3					
8	181.6	176.7	183.2	172.5	176.5					
9	162.7	156.8	161.7	159.4	162.4					
11	35.5	37.1	32.6	36.9	29.8	3.50	3.69	3.11	3.71	3.08
12	49.8	57.2	54.4	58.4	53.1	2.66	3.55	2.95	3.57	2.93
CMAE			1.5		3.1			0.23		0.25
DP4		100.0%		0.0%			85.3%		14.7%	

**Scheme 1:**
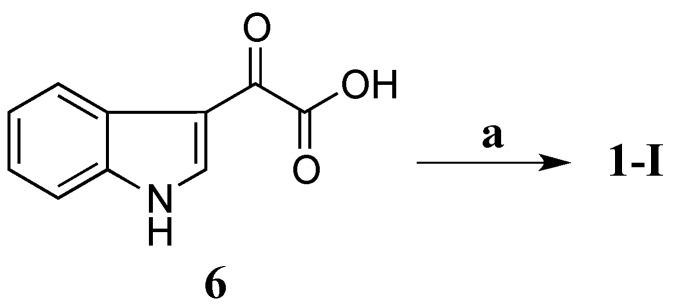
Synthesis of **1**. (**a**) Taurine, EDCI, HOBt, DMF, rt, 21 h, 64%.

Compound **1-I** was synthesized by coupling 3-indoleglyoxylic acid (**6**) with taurine (Scheme 1) in the presence of *N*-(3-dimethylaminopropyl)-*N′*-ethylcarbodiimide (EDCI), *N*-hydroxybenzotriazole (HOBt) and dimethylformamide (DMF) at room temperature (rt) for 21 h (64% yield) [26]. Both ^1^H and ^13^C NMR data of the synthetic **1-I** completely matched those of the natural product **1** (Table 3) confirming that **1-I** was the structure of **1**. Therefore, compound **1** was defined as a new alkaloid with a trivial name stolonine A.

**Table 3:** ^1^H and ^13^C NMR chemical shifts of natural and synthetic products of **1**–**3** ^a^.

Position	1				2				3			
	Natural Product ^a^		Synthetic Product ^b^		Natural Product ^a^		Synthetic Product ^b^		Natural Product ^a^		Synthetic Product ^b^	
	δ_C_	δ_H_ (*J* in Hz)	δ_C_	δ_H_ (*J* in Hz)	δ_C_	δ_H_ (*J* in Hz)	δ_C_^a^	δ_H_ (*J* in Hz)	δ_C_	δ_H_ (*J* in Hz)	δ_C_	δ_H_ (*J* in Hz)
1		12.20, br.s		12.19, br.s					131.3	8.95, s	131.3	8.95, s
2	138.5	8.82, d (3.0)	138.5	8.82, d (3.0)	150.1		150.1					
3	112.1		112.1		118.6	8.15, d (8.4)	118.6	8.15, d (8.5)	138.0		138.0	
3a	126.2		126.2									
4	121.3	8.22, dd (1.8, 7.2)	121.3	8.22, d (7.5)	137.8	8.55, d (8.4)	137.8	8.55, d (8.5)	114.0	8.93, s	114.1	8.93, s
4a					129.2		129.2		128.9		129.0	
4b									120.7		120.7	
5	122.4	7.24, dt (1.8, 7.2)	122.4	7.24, t (7.5)	128.7	8.08, d (7.8)	128.7	8.08, d (8.0)	122.3	8.39, d (7.8)	122.3	8.39, d (8.0)
6	123.3	7.26, dt (1.8, 7.2)	123.3	7.26, t (7.5)	128.0	7.71, t (7.2, 7.8)	128.0	7.71, t (8.0)	120.3	7.35, t (7.2, 7.8)	120.3	7.35, t (7.5, 8.0)
7	112.5	7.52, dd (1.8, 7.2)	112.5	7.52, d (7.5)	130.4	7.87, t (7.2, 7.8)	130.4	7.87, t (8.0)	129.2	7.64, t (7.2, 7.8)	129.2	7.64, t (7.5, 8.0)
7a	136.2		136.2									
8	181.6		181.6		128.1	8.06, d (7.8)	128.1	8.06, d (8.0)	112.5	7.70, d (7.8)	112.5	7.70, d (8.0)
8a					146.0		146.0		141.6		141.6	
9	162.7		162.7		163.5		163.5			12.11, s		12.10, s
9a									136.7		136.6	
10		8.78, t (5.4)		8.78, t (5.4)		9.29, t (5.4)		9.29, t (5.0)	163.3		163.3	
11	35.5	3.50, dd (6.0, 6.6)	35.5	3.50, dd (6.0, 6.5)	35.9	3.65, dd (6.0, 6.6)	35.9	3.65, dd (5.5, 6.5)		9.15, t (5.4)		9.15, t (5.5)
12	49.8	2.66, t (6.6)	49.8	2.66, t (6.5)	50.1	2.72, t (6.6)	50.1	2.72, t (6.5)	35.9	3.66, dd (5.4, 6.6)	35.9	3.66, dd (5.5, 6.0)
13									50.3	2.72, t (6.6)	50.3	2.72, t (6.0)

^a^ ^1^H NMR at 600 MHz at 30 °C referenced to residual DMSO solvent (δ_H_ 2.50 ppm) and ^13^C NMR at 150 MHz at 30 °C referenced to residual DMSO solvent (δ_C_ 39.52 ppm); ^b^ ^1^H NMR at 500 MHz at 30 °C referenced to residual DMSO solvent (δ_H_ 2.50 ppm) and ^13^C NMR at 125 MHz at 30 °C referenced to residual DMSO solvent (δ_C_ 39.52 ppm).

Stolonine B (**2**) was purified as a white amorphous solid. A molecular ion [M − H]^−^ at *m*/*z* 279.0441 in the (−)-HRESIMS spectrum indicated that **2** had a molecular formula C_12_H_12_N_2_O_4_S. A ^1^H NMR spectrum of **2** displayed one exchangeable proton (δ_H_ 9.29 ppm), six aromatic protons (δ_H_ 8.55, 8.15, 8.08, 8.06, 7.87 and 7.71 ppm) and two methylenes (δ_H_ 3.65 and 2.72 ppm). ^13^C NMR combined with gHSQCAD spectra indicated that **2** contained nine aromatic carbons including six tertiary carbons (δ_C_ 137.8, 130.4, 128.7, 128.1, 128.0 and 118.6 ppm) and three quaternary carbons (δ_C_ 150.1, 146.0 and 129.2 ppm), one carbonyl (δ_C_ 163.5 ppm) and two methylenes (δ_C_ 50.1 and 35.9 ppm) (Table 4). Compared to **1**, compound **2** displayed similar COSY and HMBC correlations from the two methylene protons resulting in the assignment of a taurine moiety. A 1,2-disubstituted benzene ring was deduced on the basis of *J* coupling constants and COSY correlations of H-5 (δ_H_ 8.08, d, 7.8 Hz), H-6 (δ_H_ 7.71, t, 7.2, 7.8 Hz), H-7 (δ_H_ 7.87, t, 7.2, 7.8 Hz) and H-8 (δ_H_ 8.06, d, 7.8 Hz). Two doublet aromatic protons H-3 (δ_H_ 8.15 ppm) and H-4 (δ_H_ 8.55 ppm) coupling together with a *J* coupling value of 8.4 Hz showed HMBC correlations from H-3 to C-4a (δ_C_ 129.2 ppm) and from H-4 to C-5 (δ_C_ 128.7 ppm) and C-8a (δ_C_ 146.0 ppm) facilitating a connection from **c** to **b** at C-4. Two quaternary carbons C-2 (δ_C_ 150.1 ppm) and C-8a (δ_C_ 146.0 ppm) were linked through a nitrogen atom N-1 based on their downfield resonances, which are characteristic for the imine carbons [27]. According to a HMBC correlation from H-4 to the imine C-2, C-2 was attached to C-3 leading to the establishment of a quinoline ring system.

**Table 4:** NMR data for **2** in DMSO-*d_6_* ^a^.

Position	δ_C_	δ_H_ (*J* in Hz)	gCOSY	gHMBCAD
2	150.1			
3	118.6	8.15, d (8.4)	4	4a
4	137.8	8.55, d (8.4)	3	2, 5, 8a
4a	129.2			
5	128.7	8.08, d (7.8)	6	4, 7, 8a
6	128.0	7.71, t (7.2, 7.8)	5, 7	4a, 8
7	130.4	7.87, t (7.2, 7.8)	6, 8	5, 8a
8	128.1	8.06, d (7.8)	7	4a, 6
8a	146.0			
9	163.5			
10		9.29, t (5.4)	11	
11	35.9	3.65, dd (5.4, 6.6)	10, 12	9, 12
12	50.1	2.72, t (6.6)	11	11

^a^ ^1^H NMR at 600 MHz referenced to residual DMSO solvent (δ_H_ 2.50 ppm) and ^13^C NMR at 150 MHz referenced to residual DMSO solvent (δ_C_ 39.52 ppm).

**Scheme 2:**
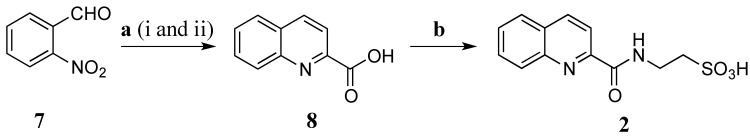
Synthesis of **2**. (**a**) (i) Fe, HCl, EtOH, reflux, 40 min; (ii) methyl pyruvate, KOH, reflux, 1.5 h, 55% (**b**) Taurine, EDCI, HOBt, DMF, rt, 48 h, 75%.

Due to lack of HMBC correlations from H-3 (δ_H_ 8.15 ppm) to C-9 (δ_C_ 163.5 ppm) or from H-10 (δ_H_ 9.29 ppm) to C-2 to obtain unequivocal evidence of the final structure, total synthesis of **2** was undertaken to verify the structure assignment (Scheme 2). *o*-Nitrobenzaldehyde (**7**) was reduced with 4.5 equivalents (eq.) of iron powder in the presence of 0.05 mol of HCl (aqueous, aq.) in ethanol (EtOH) under reflux in 40 min. Methyl pyruvate and potassium hydroxide powder were then added and the condensation reaction was under reflux in additional 1.5 h to obtain quinoline-2-carboxylic acid (**8**) (55% yield) [28]. This compound was coupled with taurine using EDCI/HOBt in DMF at rt for 48 h to produce **2** with a yield of 75%. The NMR data of the synthetic compound **2** (Table 3) was identical to that of the natural product **2** confirming the structure assignment of **2** as a new taurine amide. Thus, the structure of **2**, stolonine B, was established.

**Figure 4:**
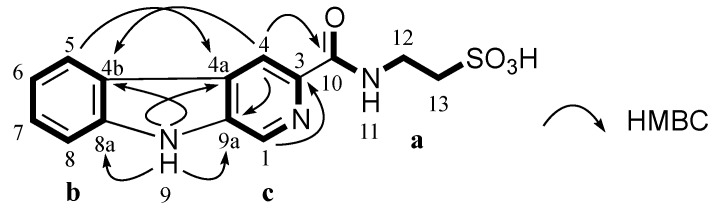
Partial structures (**a**, **b** and **c**) of **3** and their key HMBC correlations.

Stolonine C (**3**) was isolated as a white amorphous solid. Its (−)-HRESIMS spectrum showed a signal for [M − H]^−^ at *m*/*z* 318.0550 indicating a molecular formula C_14_H_13_N_3_O_4_S to be assigned to **3**. A ^13^C NMR spectrum combined with 2D NMR data indicated that compound **3** had 11 aromatic carbons including six tertiary carbons (δ_C_ 132.2, 128.2, 122.3, 120.0, 113.9 and 112.3 ppm), five quaternary carbons (δ_C_ 141.1, 139.6, 137.1, 128.7 and 121.0 ppm), one carbonyl (δ_C_ 164.3 ppm) and two methylenes (δ_C_ 50.4 and 35.7 ppm) (Table 5). COSY and HMBC correlations confirmed **3** had a taurine moiety (**a**, Figure 4) and a 1,2-disubstituted benzene ring (**b**, Figure 4), which were similar to those in **1** and **2**. HMBC correlations from a singlet proton H-1 (δ_H_ 8.89 ppm) to C-3 (δ_C_ 139.6 ppm), C-4a (δ_C_ 128.7 ppm) and C-9a (δ_C_ 137.1 ppm) and from a singlet proton H-4 (δ_H_ 8.84 ppm) to C-9a indicated the presence of a 2,4,5-trisubstituted pyridine ring (**c**, Figure 4). HMBC correlations from an exchangeable proton H-9 (δ_H_ 11.96 ppm) to C-4a, C-9a, C-4b (δ_C_ 121.0 ppm) and C-8a (δ_C_ 141.1 ppm) supported the connection of **b** to **c** forming a β-carboline ring system. A HMBC key correlation from H-4 to C-10 (δ_C_ 164.3 ppm) allowed the connection of **c** to **a** at C-3 (δ_C_ 139.6 ppm). Therefore, the structure of stolonine C (**3**) was determined as shown in Figure 4.

**Table 5:** NMR data for **3** in DMSO-*d_6_*.

Position	δ_C_^a^	δ_H_ (*J* in Hz) ^a^	δ_C_^b,c^	δ_H_ (*J* in Hz) ^b^	gCOSY ^b^	gHMBCAD ^b^
1	132.2	8.89, s	131.3	8.95, s		3, 4a, 9a
3	139.6		138.0			
4	113.9	8.84, s	114.0	8.93, s		4b, 9a, 10
4a	128.7		128.9			
4b	121.0		120.7			
5	122.3	8.39, d (8.1)	122.3	8.39, d (7.8)	6	4a, 7, 8a
6	120.0	7.30, t (7.2, 8.1)	120.3	7.35, t (7.2, 7.8)	5, 7	4b, 8
7	128.2	7.59, t (7.2, 8.1)	129.2	7.64, t (7.2, 7.8)	6, 8	5, 8a
8	112.3	7.65, d (8.1)	112.5	7.70, d (7.8)	7	4b, 6
8a	141.1		141.6			
9		11.96, s		12.11, s		4a, 4b, 8a, 9a
9a	137.1		136.7			
10	164.3		163.3			
11		9.09, t (5.4)		9.15, t (5.4)	12	
12	35.7	3.64, dd (5.4, 6.3)	35.9	3.66, dd (5.4, 6.6)	11, 13	10, 13
13	50.4	2.70, t (6.3)	50.3	2.72, t (6.6)	12	12

^a^ ^1^H NMR at 900 MHz at 25 °C referenced to residual DMSO solvent (δ_H_ 2.50 ppm) and ^13^C NMR at 225 MHz at 25 °C referenced to residual DMSO solvent (δ_C_ 39.52 ppm); ^b^ ^1^H NMR at 600 MHz at 30 °C referenced to residual DMSO solvent (δ_H_ 2.50 ppm) and ^13^C NMR at 150 MHz at 30 °C referenced to residual DMSO solvent (δ_C_ 39.52 ppm); ^c^ ^13^C chemical shifts obtained from correlations observed in gHSQCAD and gHMBCAD spectra.

**Scheme 3:**
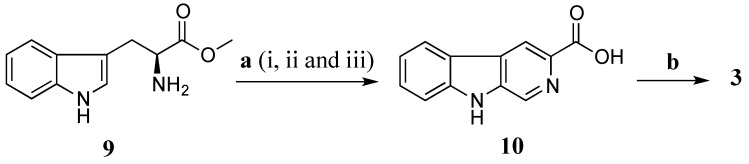
Synthesis of **3**. (**a**) (i) HCHO, MeOH, HCl 0.1 N, rt, 16 h; (ii) MnO_2_, C_6_H_6_, reflux, 5 h; (iii) NaOH 20%, MeOH, reflux, 45 min (10% in three steps); (**b**) Taurine, EDCI, HOBt, DMF, rt, 16 h, 40%.

Total synthesis of **3** was performed using l-tryptophan methyl ester (**9**) as a starting material (Scheme 3). The l-tryptophan methyl ester was treated with formaldehyde (37% aqueous) in a mixture of MeOH and HCl 0.1 N (ratio 10:1) at rt for 16 h to give methyl (3*S*)-1,2,3,4-tetrahydro-β-carboline-3-carboxylate [29]. The crude methyl (3*S*)-1,2,3,4-tetrahydro-β-carboline-3-carboxylate was further oxidized by activated manganese (IV) oxide (MnO_2_) in benzene (C_6_H_6_) under reflux for 5 h yielding a crude methyl β-carboline-3-carboxylate [30]. This ester was then hydrolysed in a mixture of aq. NaOH 20% and MeOH (ratio 1:4) to provide β-carboline-3-carboxylic acid (**10**) (10% yield in three steps) [30]. Amide coupling of **10** and taurine was performed using EDCI/HOBt in DMF at rt in 16 h to produce **3** with a yield of 40%. The synthetic product **3** had superimposable ^1^H and ^13^C NMR data with stolonine C (**3**) confirming its structure assignment (Table 3).

**Scheme 4:**
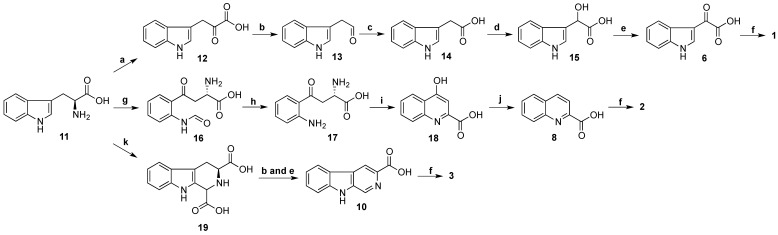
Biosynthesis of stolonines A–C (**1**–**3**). **a**: transaminase; **b**: decarboxylase; **c**: aldehyde dehydrogenase; **d**: hydroxylase; **e**: dehydrogenase; **f**: taurine + non-ribosomal peptide synthase or acyl-CoA:amino acid *N*-acyltransferase 1; **g**: tryptophan 2,3-dioxygenase or indoleamine 2,3-dioxygenase; **h**: kynurenine formamidase or arylformamidase; **i**: kynurenine aminotransferases; **j**: reductase; **k**: glyoxylic acid.

Biosynthesis of stolonines A–C (**1**–**3**) is shown in Scheme 4. Tryptophan (**11**) has been known as a precursor of 3-indoleglyoxylic acid (**6**), quinoline-2-carboxylic acid (**8**) and β-carboline-3-carboxylic acid (**10**) [31]. The acids **6**, **8** and **10** undergo amide bond formation with taurine either by non-ribosomal peptide synthase [32,33] or by acyl-CoA:amino acid *N*-acyltransferase 1 [34,35] to produce stolonines A–C (**1**–**3**).

The two known compounds, 11-hydroxyascididemin (**4**) and cnemidine A (**5**), were identified by NMR comparisons with those in the literature [11].

**Figure 5:**
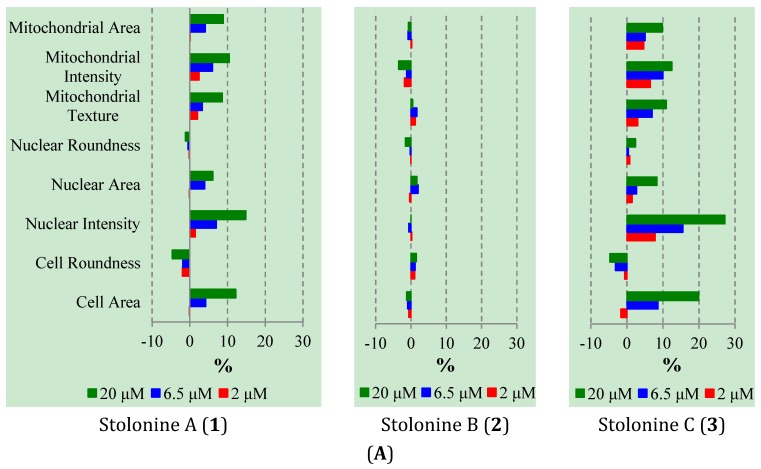

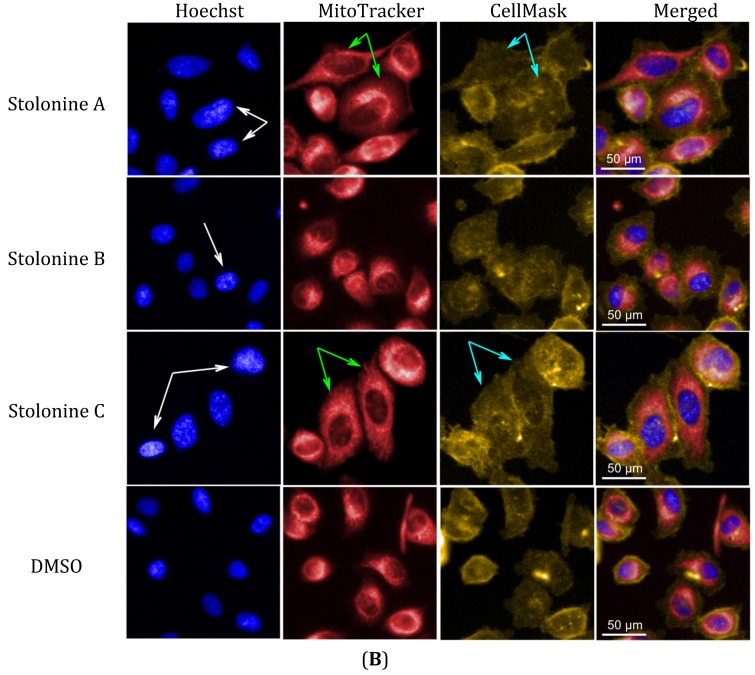
(**A**) Phenotypic profiles for cell proliferation study of **1**–**3** (results were normalized by a vehicle DMSO); (**B**) Representative images of the PC3 cells treated with 20 μM of **1**–**3** and DMSO in three channels: Hoechst 33,342 (Blue), MitoTracker (Red) and CellMask (Yellow) (scale bar: 50 μm); chromatin condensation (white arrow), mitochondrial texture elongation (green arrow) and larger cell size (cyan arrow) compared with vehicle DMSO.

Cytotoxic evaluation of compounds **1**–**3** against PC3 cells using the Alamar blue assay indicated that these compounds inhibited the growth of PC3 cells at only 19%, 14%, and 26%, respectively, at their maximum tested concentration of 20 μM. In order to explore whether these compounds have any effect on cellular organelles in PC3 cells, an immunofluorescence assay with three markers for cell membrane, nuclei, and mitochondria was performed. A high-content imaging system was used to image and analyze the data. Compared to vehicle, compounds **1** and **3** had effects on cell morphology, nuclei and mitochondria while compound **2** showed no or very weak effects (Figure 5A). In general, the influence of **1** and **3** on PC3 cells was similar and clearly observed in cell morphology, nuclear, and mitochondrial intensities as well as mitochondrial texture in a dose-dependent manner. In particular, cells treated with **1** and **3** increased cell size and induced cells to become longer shown by the increase in cell, mitochondrial and nuclear area, and the decrease in cell roundness. Compounds **1** and **3** also caused mitochondrial texture to become larger and elongated compared to **2** and DMSO. The increase in nuclear and mitochondrial intensities, which displayed brighter blue-whitish and brighter red-whitish fluorescent appearance respectively in the images (Figure 5B), indicated that the nuclei and mitochondria had more packed masses [36]. The brighter blue-whitish also demonstrated the condensation of chromatins [37], which has been considered as a hallmark feature of apoptotic pathway of programmed cell death [38,39]. The phenotype of nuclei suggested that compounds **1** and **3** affected the PC3 cell death via apoptosis. Moreover, at the non-toxic doses, compounds **1** and **3** also increased the cell size and induced mitochondrial texture elongation. How these compounds influence the PC3 cell morphology and mitochondria is something that warrants further investigation.

## 3. Experimental Section

### 3.1. General Experimental Procedures

UV spectra were recorded on a CAMSPEC M501 UV/V is spectrophotometer. NMR spectra were recorded at 30 °C on Varian Inova 500 and 600 MHz and on Bruker 900 MHz spectrometers. The ^1^H and ^13^C chemical shifts were referenced to the DMSO-*d_6_* solvent peaks at δ_H_ 2.50 and δ_C_ 39.52 ppm. Standard parameters were used for the 2D NMR spectra obtained, which included gCOSY, gHSQCAD (^1^*J*_CH_ = 140 Hz), gHMBCAD (*^n^J*_CH_ = 8 Hz), and ROESY. Low-resolution mass spectra were acquired using a Mariner TOF mass spectrometer (Applied Biosystems Pty Ltd, Melbourne, Australia.). High-resolution mass measurement was acquired on a Bruker Solarix 12 Tesla Fourier transform mass spectrometer, fitted with an Apollo API source. For the HPLC isolation, a Water 600 pump equipped with a Water 966 PDA detector and Gilson 715 liquid handler were used. A Betasil C18 column (5 μm, 21.2 × 150 mm) and Hypersil BDS C_18_ column (5 μm, 10 × 250 mm) were used for semi-preparative HPLC. A Phenomenex Luna C_18_ column (3 μm, 4.6 × 50 mm) was used for LC-MS controlled by MassLynx 4.1 software (Waters, Milford, MA, USA). All solvents used for extraction and chromatography were HPLC grade from RCI Labscan or Burdick & Jackson (Lomb Scientific, Sydney, Australia), and the H_2_O used was ultrapure water (Arium^®^ proVF, Sartorius Stedim Biotech, Melbourne, Australia).

### 3.2. Animal Material

A specimen of the tunicate *C. stolonifera* (phylum Tunicata, class Ascidiacea, order Stolidobranchia, family Styelidae) was collected at the depth of 15 m, at Peel Island, Myora Light, North Stradbroke Island, Queensland, Australia in 2005. A voucher specimen (G322208) has been stored at the Queensland Museum, South Brisbane, Queensland, Australia. Material was collected under Moreton Bay Marine Park Permit Number QS2005/CVL588 and Queensland Fisheries Service General Fisheries Permit Number PRM02988B issued to the Queensland Museum.

### 3.3. Extraction and Isolation

The freeze-dried and ground tunicate *C. stolonifera* (19 g) was extracted exhaustively with hexane (250 mL), DCM (250 mL) and MeOH (2 × 250 mL), respectively. The DCM and MeOH extracts were combined and the solvents were evaporated to yield a yellow residue (2.2 g). This extract was pre-adsorbed onto C_18_ (1 g) and packed dry into a small cartridge, which was connected to a C_18_ preparative HPLC column (5 μm, 21.2 × 150 mm). A linear gradient from 100% H_2_O (0.1% TFA) to 100% MeOH (0.1% TFA) was performed over 60 min at a flow rate of 9 mL/min and 60 fractions (1.0 min each) were collected. Pure cnemidine A (**5**, 3.5 mg, 0.018% dry wt) and 11-hydroxyascididemin (**4**, 4.1 mg, 0.021% dry wt) were obtained in fractions 28, 29 and 32 respectively. Fraction 19 was further chromatographed on a Hypersil C_18_ HPLC column (5 μm, 10 × 250 mm) from 10% MeOH (0.1% TFA)/90% H_2_O (0.1% TFA) to 25% MeOH (0.1% TFA)/75% H_2_O (0.1% TFA) at a flow rate of 4 mL/min in 30 min to yield stolonine C (**3**, 0.1 mg, 0.0021% dry wt) in fraction 14 and stolonine A (**1**, 0.9 mg, 0.0047% dry wt) in fractions 17–18. Fractions 20 and 21 in the first chromatography were combined and purified using the Hypersil C_18_ HPLC column (5 μm, 10 × 250 mm) from 10% MeOH (0.1% TFA)/90% H_2_O (0.1% TFA) to 40% MeOH (0.1% TFA)/60% H_2_O (0.1% TFA) at a flow rate of 4 mL/min in 45 min to obtain stolonine C (**3**, 0.3 mg, 0.0021% dry wt in total) in fraction 33 and stolonine B (**2**, 1.2 mg, 0.0063% dry wt) in fractions 34–36.

Stolonine A (**1**): white, amorphous solid; UV (MeOH) λ_max_ (log ε) 210 (3.8), 252 (3.5) and 325 (3.3) nm; IR (film) ν_max_ 3307, 1681, 1205, 1049 and 802 cm^−1^; ^1^H (600 MHz, DMSO-*d_6_*) and ^13^C (150 MHz, DMSO-*d_6_*) NMR data are summarized in Table 1; (−)-HRESIMS *m*/*z* 295.0390 [M − H]^−^ (calcd for [C_12_H_11_N_2_O_5_S]^−^, 295.0394, Δ −1.4 ppm).

Stolonine B (**2**): white, amorphous solid; UV (MeOH) λ_max_ (log ε) 210 (3.8) and 240 (3.9) nm; IR (film) ν_max_ 3386, 1684, 1204 and 1066 cm^−1^; ^1^H (600 MHz, DMSO-*d_6_*) and ^13^C (150 MHz, DMSO-*d_6_*) NMR data are summarized in Table 3; (−)-HRESIMS *m*/*z* 279.0441 [M − H]^−^ (calcd for [C_12_H_11_N_2_O_4_S]^−^, 279.0445, Δ −1.4 ppm).

Stolonine C (**3**): white, amorphous solid; UV (MeOH) λ_max_ (log ε) 210 (4.1) and 272 (3.8) nm; IR (film) ν_max_ 3418, 1682, 1197 and 1034 cm^−1^; ^1^H (600 MHz, DMSO-*d_6_*) and ^13^C (150 MHz, DMSO-*d_6_*) NMR data are summarized in Table 4; (−)-HRESIMS *m*/*z* 318.0550 [M − H]^−^ (calcd for [C_14_H_12_N_3_O_4_S]^−^, 318.0554, Δ −1.3 ppm).

Synthetic stolonine A (**1**): To a solution of taurine (12 mg, 0.096 mmol) in dry DMF (1 mL) was added HOBt (26 mg, 0.193 mmol) and the reaction mixture was stirred for 15 min at rt. The reaction mixture was then cooled to 0 °C, EDCI (37 mg, 0.193 mmol) was added and continued stirring for 30 min at 0 °C. To this mixture was then added 3-indoleglyoxylic acid (**6**, 18 mg, 0.095 mmol) and the mixture was stirred for 21 h at rt. The crude product was concentrated *in vacuo* and separated by RP-HPLC (MeOH, H_2_O, 0.1% TFA) to obtain the synthetic stolonine A (18 mg, 64%); IR (film) ν_max_ 3371, 1681, 1205, 1047, 802 and 749 cm^−1^; ^1^H (600 MHz, DMSO-*d_6_*) and ^13^C (150 MHz, DMSO-*d_6_*) NMR data are summarized in Table 5; (−)-HRESIMS *m*/*z* 295.0391 [M − H]^−^ (calcd for [C_12_H_11_N_2_O_5_S]^−^, 295.0394, Δ −1.0 ppm).

Quinoline-2-carboxylic acid (**8**): To a solution of *o*-nitrobenzaldehyde (**7**, 302 mg, 2 mmol) in ethanol (5 mL) was added iron powder (504 mg, 9 mmol), followed by 0.1 N HCl (2 mL, 0.2 mmol) and the resulting mixture was vigorously stirred under reflux for 45 min. Methyl pyruvate (200 μL, 2 mmol) and powder KOH (135 mg, 2.4 mmol) were added slowly. The reaction mixture was stirred under reflux for 90 min and then cooled to rt. The crude product was purified by RP-HPLC (MeOH, H_2_O, 0.1% TFA) to yield **8** (190 mg, 55% in two steps); ^1^H NMR (500 MHz, DMSO-*d_6_*) δ 8.56 (1H, d, *J* = 8.5 Hz), 8.16 (1H, d, *J* = 8.0 Hz), 8.11 (1H, d, *J* = 8.5 Hz), 8.09 (1H, d, *J* = 8.0 Hz), 7.88 (1H, t, *J* = 8.0 and 7.5 Hz) and 7.74 (1H, t, *J* = 8.0 and 7.5 Hz); ^13^C NMR (125 MHz, DMSO-*d_6_*) δ 166.2 (COOH), 148.5 (C), 146.6 (C), 137.6 (CH), 130.5 (CH), 129.5 (CH), 128.7 (C), 128.5 (CH), 127.9 (CH) and 120.6 (CH). (+)-LRESIMS *m*/*z* 174.

Synthetic stolonine B (**2**): To a solution of taurine (13 mg, 0.104 mmol) in dry DMF (1 mL) was added HOBt (28 mg, 0.207 mmol) and the reaction mixture was stirred for 15 min at rt. The reaction mixture was then cooled to 0 °C, EDCI (40 mg, 0.209 mmol) was added and continued stirring for 30 min at 0 °C. To this mixture was then added compound **8** (18 mg, 0.104 mmol) and the mixture was stirred for 48 h at rt. The pure synthetic stolonine B was purified by RP-HPLC (MeOH, H_2_O, 0.1% TFA) to obtain 21.8 mg (75% yield); IR (film) ν_max_ 3383, 1677, 1201 and 1064 cm^−1^; ^1^H (600 MHz, DMSO-*d_6_*) and ^13^C (150 MHz, DMSO-*d_6_*) NMR data are summarized in Table 5; (−)-HRESIMS *m/z* 279.0446 [M − H]^−^ (calcd for [C_12_H_11_N_2_O_4_S]^−^, 279.0445, Δ 0.4 ppm).

β-carboline-3-carboxylic acid (**10**): Formaldehyde (37%, 2 mL) was added to l-tryptophan methyl ester hydrochloride (**9**) (2.54 g, 10 mmol) in aqueous MeOH (10 mL, MeOH-H_2_O (10:1)). The resulting mixture was stirred at rt for 16 h. The reaction mixture was evaporated to dryness *in vacuo* and oxidized by activated MnO_2_ (2.5 g, 28.7 mmol) in benzene under reflux for 5 h. The hot solution was filtered through a bed of C_18_ to remove the MnO_2_ and the filter cake was washed with hot benzene. The crude product was suspended in a mixture of aq. NaOH 20% and MeOH (ratio 1:4) and heated to reflux for 45 min. The reaction was cooled to rt, evaporated to dryness *in vacuo* and then loaded onto RP-HPLC (MeOH, H_2_O, 0.1% TFA) yielding **10** (215 mg, 10% in three steps). ^1^H NMR (600 MHz, DMSO-*d_6_*) δ 12.07 (1H, s), 8.98 (1H, s), 8.92 (1H, s), 8.39 (1H, d, *J* = 8.4 Hz), 7.67 (1H, d, *J* = 8.4 Hz), 7.60 (1H, t, *J* = 7.2, 7.8 Hz), 7.31 (1H, t, *J* = 7.2, 7.8 Hz); ^13^C NMR (150 MHz, DMSO-*d_6_*) δ 166.8 (COOH), 141.1 (C), 137.4 (2xC), 133.1 (CH), 128.7 (CH), 127.8 (C), 122.2 (CH), 120.9 (C), 120.1 (CH), 117.2 (CH), 112.3 (CH). (+)-LRESIMS *m*/*z* 213.

Synthetic stolonine C (**3**): To a solution of taurine (5 mg, 0.040 mmol) in dry DMF (1 mL) was added HOBt (13.5 mg, 0.1 mmol) and the reaction mixture was stirred for 15 min at rt. The reaction mixture was then cooled to 0 °C, EDCI (19 mg, 0.099 mmol) was added and continued stirring for 30 min at 0 °C. To this mixture was then added **10** (8 mg, 0.038 mmol) and the mixture was stirred for 16 h at rt. The crude product was concentrated *in vacuo* and separated by RP-HPLC (MeOH, H_2_O, 0.1% TFA) to obtain the synthetic stolonine C (4.8 mg, 40%); IR (film) ν_max_ 3419, 1683, 1198 and 1034 cm^−1^; ^1^H (600 MHz, DMSO-*d_6_*) and ^13^C (150 MHz, DMSO-*d_6_*) NMR data are summarized in Table 5; (−)-HRESIMS *m*/*z* 318.0556 [M − H]^−^ (calcd for [C_14_H_12_N_3_O_4_S]^−^, 318.0554, Δ 0.6 ppm).

### 3.4. Computational Details

Molecular mechanics calculations were performed using Macromodel [40] interfaced to the Maestro program [41]. All conformational searches used the MMFFs force field. Twenty-one conformers of **1-I** and six conformers of **1-II** within a relative energy of 2 kcal/mol were found. The geometries of these conformers were subsequently optimized at DFT level with the B3LYP functional and 6-31G(d) basis set using Jaguar [42]. Single point calculations in DMSO with the mPW1PW91 functional and the same basis set were employed using Jaguar [42] to provide the shielding constant of carbon and proton nuclei. Meanwhile, the same procedure was applied on tetramethylsilane (TMS). Final ^1^H and ^13^C chemical shifts were obtained as the results of the Boltzmann weighted average. The theoretical chemical shifts were calculated according to a below equation.

(1) $${\text{δ}}_{calc}^{x}={\text{σ}}_{TMS}-{\text{σ}}^{x}$$

where ${\text{δ}}_{calc}^{x}$ is the calculated shift for nucleus *x* (in ppm); ${\text{σ}}^{x}$ is the shielding constant for nucleus *x*; ${\text{σ}}_{TMS}$ is the shielding constant for the carbon in TMS ${\text{σ}}_{TMS}$ = 194.6867 ppm and for the proton in TMS ${\text{σ}}_{TMS}$ = 32.0845 ppm.

Statistical parameters were used to quantify the agreement between experimental and calculated data:

- The slope (a), the intercept (b) and the correlation coefficient (*R^2^*) were determined from a plot of ${\text{δ}}_{calc}$ against ${\text{δ}}_{exp}$ for each particular compound.
- Systematic errors during the shift calculation were removed by empirical scaling according to ${\text{δ}}_{scaled}=\left({\text{δ}}_{calc}-b\right)/a$
- The corrected mean absolute error (CMAE) was defined as $\sum_{i=1}^{n}\left|{\text{δ}}_{scaled}-{\text{δ}}_{exp}\right|/n$
- DP4 parameter was calculated by using the online applet [43].

### 3.5. Cytotoxicity Assay

Human prostate adenocarcinoma cells (PC3) and human neonatal foreskin fibroblast (noncancer cells, NFF) were grown in media RPMI-1640 (Life Technologies, Grand Island, NY, USA) supplemented with 10% foetal bovine serum (FBS) (Life Technologies, Grand Island, NY, USA). Cells were grown under 5% CO_2_ in a humidified environment at 37 °C. Fifty microliters of media containing 500 cells were added to a 384 well microtiter plate (Perkin Elmer, Shelton, CT, USA, part number: 6,007,660) containing 0.2 μL of a compound. Final compound concentrations tested were 20, 6.5, 2, 0.65, 0.2, 0.065, 0.020 and 0.0065 μM (final DMSO concentration of 0.4%). Each concentration in media was tested in triplicate and in two independent experiments. Cells and compounds were then incubated in 72 h at 37 °C, 5% CO_2_ and 80% humidity. Cell proliferation was measured with the addition of 10 μL of a 60% Alamar blue (Invitrogen, Carlsbad, CA, USA) solution in media to each well of the microtiter plate to give a final concentration of 10% Alamar blue. The plates were incubated at 37 °C, 5% CO_2_ and 80% humidity within 24 h. The fluorescence of each well was read at excitation 535 nm and emission 590 nm on the Perkin Elmer EnVision Multilabel Reader 2104. Eight-point concentration response curves were then analyzed using non-linear regression and IC_50_ values determined by using GraphPad Prism 5 (GraphPad Software Inc., La Jolla, CA, USA). Paclitaxel and doxorubicin were used during each screening as positive control compounds.

### 3.6. Immunofluorescence Staining

Cells (2000 cells/well) were allowed to seed in a 96-well plate (CellCarrier, Perkin Elmer, Shelton, CT, USA, part number: 6,005,558) containing 0.4 μL of a compound at room temperature for 30 min and then placed under 5% CO_2_ in a humidified environment at 37 °C. After 72 h incubation, 100 μL of a solution containing 1/1000 Hoechst 33,342 (Molecular Probes, Invitrogen, Carlsbad, CA, USA), 1 μM MitoTracker Deep Red (Molecular Probes, Invitrogen, Carlsbad, CA, USA) and 1/1000 CellMask Plasma Membrane (Molecular Probes, Invitrogen, Carlsbad, CA, USA) were added to each well and incubated for 30 min at 37 °C. Stained cells were washed once with PBS to remove excess stains and then replaced with 100 μL of PBS before being imaged.

### 3.7. Automated Microscopy and Image Analysis

Plates were imaged with an Operetta high-content wide-field fluorescence imaging system, coupled to Harmony software (Perkin Elmer, Shelton, CT, USA). Wells in a 96-well plate were captured at 25 locations per well at 20× magnification at three wavelengths (350 nm: Hoechst 33,342; 554 nm: CellMask Plasma Membrane; 644 nm: MitoTracker Deep Red). The three images were combined and analyzed using the Harmony software. The analysis protocol involved in the following steps: (1) each cell nucleus was identified using Hoechst stain; (2) the cell cytoplasm as defined from CellMask fluorescence; (3) cells that overlapped the border of the image were excluded from the analysis. Each concentration was repeated in at least two separated experiments. To obviate observer bias, the image analysis was automated using the same parameters for every image.

## 4. Conclusions

Contributing to the chemical study on the underexplored marine tunicate *Cnemidocarpa stolonifera*, three new alkaloids stolonines A–C (**1**–**3**) belonging to the taurine amide structure class were identified. Structures of these compounds were determined by NMR spectroscopy and confirmed by synthetic methods. Stolonines A–C (**1**–**3**) represent the 48–50th naturally occurring taurine amide compounds. However, this is the first time that the conjugates of taurine with 3-indoleglyoxylic acid, quinoline-2-carboxylic acid and β-carboline-3-carboxylic acid have been reported. Although compounds **1**–**3** displayed no cytotoxicity against the prostate cancer PC3 cells at the concentration of 20 μM, an immunofluorescence assay on PC3 cells revealed that compounds **1** and **3** increased cell size, induced mitochondrial texture elongation and affected the PC3 cell death via apoptotic pathway*.* Further investigation would be necessary to understand their mechanism of action on PC3 cells.

## Supplementary Materials

IR, 1D and 2D NMR spectra of stolonines A–C (**1**–**3**) and other synthetic compounds (**8** and **10**); and calculated ^13^C and ^1^H NMR chemical shifts for each conformer of compounds **1-I** and **1-II**.
