# Supplementary material for: Isolation and Total Synthesis of Stolonines A–C, Unique Taurine Amides from the Australian Marine Tunicate Cnemidocarpa stolonifera
Source: Mar Drugs. 2015 Jul 22;13(7):4556–75. doi: 10.3390/md13074556 (PMC4515633; doi:10.3390/md13074556)
Supplement: Supplementary file 1 [file marinedrugs-13-04556-s001.docx]

Supplementary Information

**Figure S1.** ^1^H NMR spectrum for stolonine A (**1**) in DMSO-*d_6_* at 600 MHz and 30 °C.

**Figure S2.** ^13^C NMR spectrum for stolonine A (**1**) in DMSO-*d_6_* at 600 MHz and 30 °C.

**Figure S3.** gHSQCAD spectrum for stolonine A (**1**) in DMSO-*d_6_* at 600 MHz and 30 °C.

**Figure S4.** gCOSY spectrum for stolonine A (**1**) in DMSO-*d_6_* at 600 MHz and 30 °C.

**Figure S5.** gHMBCAD spectrum for stolonine A (**1**) in DMSO-*d_6_* at 600 MHz and 30 °C.

**Figure S6.** IR spectrum for stolonine A (**1**).

**Figure S7.** ^1^H NMR spectrum for stolonine B (**2**) in DMSO-*d_6_* at 600 MHz and 30 °C.

**Figure S8.** ^13^C NMR spectrum for stolonine B (**2**) in DMSO-*d_6_* at 600 MHz and 30 °C.

**Figure S9.** gHSQCAD spectrum for stolonine B (**2**) in DMSO-*d_6_* at 600 MHz and 30 °C.

**Figure S10.** gCOSY spectrum for stolonine B (**2**) in DMSO-*d_6_* at 600 MHz and 30 °C.

**Figure S11.** gHMBCAD spectrum for stolonine B (**2**) in DMSO-*d_6_* at 600 MHz and 30 °C.

**Figure S12.** ^1^H NMR spectrum for stolonine C (**3**) in DMSO-*d_6_* at 600 MHz and 30 °C.

**Figure S13.** gHSQCAD spectrum for stolonine C (**3**) in DMSO-*d_6_* at 600 MHz and 30 °C.

**Figure S14.** gCOSY spectrum for stolonine C (**3**) in DMSO-*d_6_* at 600 MHz and 30 °C.

**Figure S15.** gHMBCAD spectrum for stolonine C (**3**) in DMSO-*d_6_* at 600 MHz and 30 °C.

**Figure S16.** ^1^H NMR spectrum for stolonine C (**3**) in DMSO-*d_6_* at 900 MHz and 25 °C.

**Figure S17.** ^13^C NMR spectrum for stolonine C (**3**) in DMSO-*d_6_* at 900 MHz and 25 °C.

**Figure S18.** Comparation of ^1^H NMR spectra for natural (black) and synthetic (red) stolonine A (**1**) in DMSO-*d_6_* at 600 MHz and 30 °C.

**Figure S19.** Comparation of ^13^C NMR spectra for natural (black) and synthetic (red) stolonine A (**1**) in DMSO-*d_6_* at 600 MHz and 30 °C.

**Figure S20.** ^1^H and ^13^C NMR spectra for quinolone-2-carboxylic acid (**8**) in DMSO-*d_6_* at 600 MHz and 30 °C.

**Figure S21.** Comparation of ^1^H NMR spectra for natural (black) and synthetic (red) stolonine B (**2**) in DMSO-*d_6_* at 600 MHz and 30 °C.

**Figure S22.** Comparation of ^13^C NMR spectra for natural (black) and synthetic (red) stolonine B (**2**) in DMSO-*d_6_* at 600 MHz and 30 °C.

**Figure S23.** ^1^H and ^13^C NMR spectra for β-carboline-3-carboxylic acid (**10**) in DMSO-*d_6_* at 600 MHz and 30 °C.

**Figure S24.** Comparation of ^1^H NMR spectra for natural (black) and synthetic (red) stolonine C (**3**) in DMSO-*d_6_* at 600 MHz and 30 °C.

**Figure S25.** ^13^C NMR spectrum synthetic stolonine C (**3**) in DMSO-*d_6_* at 600 MHz
and 30 °C.


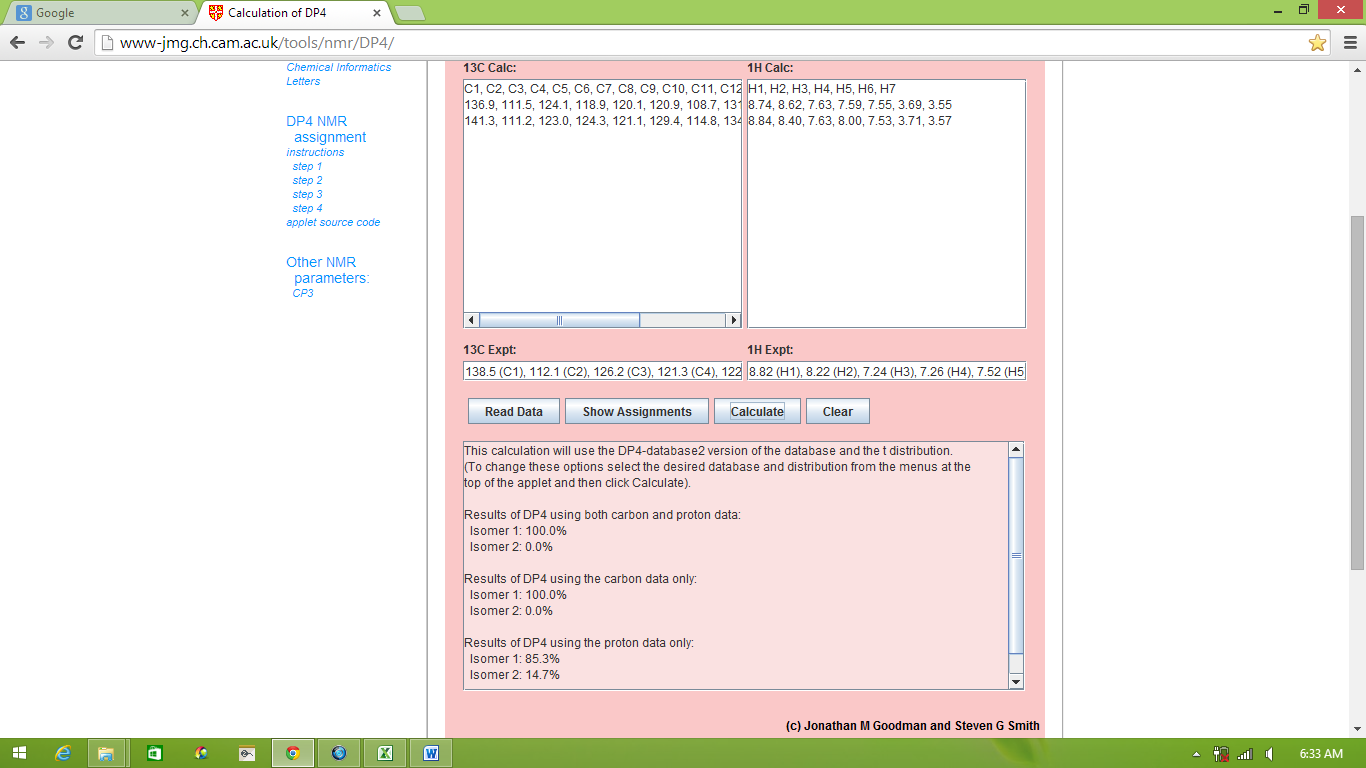


**Figure S26.** DP4 probability results of **1-I** and **1-II**.

**Table S1.** Calculated ^13^C and ^1^H NMR chemical shifts for each conformer of compound **1-I** and their Boltzmann distribution.

| **Atom** | **Conf. 1** | **Conf. 2** | **Conf. 3** | **Conf. 4** | **Conf. 5** | **Conf. 6** | **Conf. 7** | **Conf. 8** | **Conf. 9** | **Conf. 10** | **Conf. 11** | **Conf. 12** | **Conf. 13** | **Conf. 14** | **Conf. 15** | **Conf. 16** | **Conf. 17** | **Conf. 18** | **Conf. 19** | **Conf. 20** | **Conf. 21** | **Averaged** |
| --- | --- | --- | --- | --- | --- | --- | --- | --- | --- | --- | --- | --- | --- | --- | --- | --- | --- | --- | --- | --- | --- | --- |
| C2 | 133.3 | 133.9 | 132.2 | 139.6 | 136.7 | 136.4 | 136.9 | 133.6 | 136.5 | 142.4 | 136.7 | 135.2 | 137.9 | 133.5 | 136.0 | 136.7 | 133.5 | 136.3 | 137.1 | 135.4 | 138.0 | 136.9 |
| C3 | 112.8 | 111.3 | 111.8 | 114.2 | 111.6 | 111.1 | 111.5 | 111.0 | 111.4 | 113.4 | 110.6 | 111.5 | 113.4 | 112.6 | 111.3 | 112.9 | 112.5 | 110.8 | 113.4 | 112.1 | 113.2 | 111.5 |
| C3a | 121.0 | 119.7 | 121.1 | 120.9 | 123.1 | 122.1 | 124.3 | 121.8 | 124.0 | 123.4 | 123.1 | 122.1 | 120.4 | 121.2 | 122.3 | 120.3 | 120.5 | 121.8 | 121.1 | 120.7 | 119.5 | 124.1 |
| C4 | 116.7 | 114.6 | 115.0 | 118.6 | 119.1 | 118.5 | 119.0 | 118.2 | 118.9 | 117.0 | 119.3 | 118.7 | 120.1 | 116.3 | 118.4 | 118.3 | 116.1 | 118.7 | 120.5 | 117.2 | 121.1 | 118.9 |
| C5 | 118.4 | 118.5 | 118.7 | 118.7 | 120.0 | 120.0 | 120.2 | 119.6 | 120.1 | 118.2 | 120.1 | 120.2 | 119.3 | 118.4 | 119.9 | 119.2 | 118.6 | 119.9 | 119.1 | 118.9 | 119.2 | 120.1 |
| C6 | 119.8 | 119.4 | 119.7 | 119.6 | 120.9 | 120.9 | 121.0 | 120.6 | 121.0 | 118.5 | 120.9 | 121.2 | 119.9 | 119.6 | 120.8 | 119.9 | 119.7 | 120.6 | 119.7 | 119.7 | 119.9 | 120.9 |
| C7 | 109.1 | 108.8 | 109.1 | 108.7 | 108.7 | 108.8 | 108.8 | 108.8 | 108.7 | 107.2 | 108.8 | 108.7 | 108.8 | 108.9 | 108.7 | 109.0 | 109.1 | 108.7 | 108.4 | 108.8 | 108.5 | 108.7 |
| C7a | 132.1 | 131.8 | 131.9 | 133.7 | 131.3 | 131.8 | 131.9 | 131.5 | 131.9 | 133.0 | 131.3 | 132.3 | 133.1 | 132.1 | 131.9 | 132.8 | 131.7 | 131.8 | 133.1 | 132.7 | 132.7 | 131.9 |
| C8 | 183.0 | 185.0 | 184.4 | 180.8 | 178.0 | 180.9 | 176.5 | 183.0 | 177.2 | 172.0 | 177.4 | 183.8 | 179.9 | 183.4 | 181.0 | 181.4 | 183.6 | 181.1 | 179.6 | 181.7 | 179.1 | 176.7 |
| C9 | 162.8 | 166.4 | 166.8 | 157.2 | 157.7 | 160.5 | 156.4 | 163.6 | 157.3 | 158.5 | 156.5 | 162.8 | 158.0 | 164.2 | 160.8 | 160.6 | 165.3 | 160.9 | 156.6 | 163.2 | 158.1 | 156.8 |
| C11 | 39.0 | 37.9 | 40.8 | 36.2 | 37.7 | 38.3 | 36.6 | 39.6 | 38.0 | 36.7 | 37.0 | 34.9 | 36.2 | 39.7 | 39.0 | 36.2 | 40.0 | 38.3 | 36.3 | 39.2 | 36.2 | 37.1 |
| C12 | 56.2 | 59.5 | 57.5 | 59.6 | 56.3 | 56.7 | 57.9 | 58.5 | 56.0 | 56.2 | 58.9 | 64.6 | 57.0 | 56.6 | 56.0 | 58.2 | 57.1 | 57.2 | 57.5 | 58.5 | 60.4 | 57.2 |
| H2 | 8.17 | 8.28 | 8.11 | 8.51 | 8.58 | 8.03 | 8.77 | 7.80 | 8.62 | 9.37 | 8.80 | 8.08 | 8.36 | 8.17 | 7.96 | 8.35 | 8.18 | 8.02 | 8.32 | 8.35 | 8.40 | 8.74 |
| H4 | 7.44 | 7.61 | 7.65 | 8.17 | 8.51 | 8.42 | 8.65 | 8.32 | 8.60 | 8.43 | 8.49 | 8.51 | 8.37 | 7.55 | 8.39 | 7.95 | 7.55 | 8.38 | 8.39 | 7.72 | 8.83 | 8.62 |
| H5 | 7.41 | 7.46 | 7.50 | 7.41 | 7.64 | 7.61 | 7.64 | 7.58 | 7.64 | 7.54 | 7.61 | 7.62 | 7.49 | 7.43 | 7.62 | 7.47 | 7.44 | 7.61 | 7.46 | 7.44 | 7.50 | 7.63 |
| H6 | 7.50 | 7.53 | 7.56 | 7.57 | 7.58 | 7.60 | 7.60 | 7.56 | 7.60 | 7.49 | 7.58 | 7.60 | 7.54 | 7.51 | 7.60 | 7.54 | 7.53 | 7.59 | 7.52 | 7.53 | 7.51 | 7.59 |
| H7 | 7.60 | 7.66 | 7.66 | 7.58 | 7.50 | 7.56 | 7.57 | 7.52 | 7.55 | 7.19 | 7.46 | 7.56 | 7.60 | 7.61 | 7.55 | 7.61 | 7.60 | 7.50 | 7.58 | 7.61 | 7.63 | 7.55 |
| H11 | 3.82 | 3.80 | 3.78 | 3.78 | 3.71 | 3.73 | 3.75 | 3.70 | 3.57 | 3.74 | 3.58 | 3.83 | 3.88 | 3.71 | 3.75 | 3.89 | 3.73 | 3.71 | 3.82 | 3.71 | 3.96 | 3.69 |
| H12 | 3.95 | 3.77 | 4.17 | 3.46 | 3.68 | 3.73 | 3.49 | 4.04 | 3.66 | 3.59 | 3.68 | 3.47 | 3.56 | 4.09 | 3.86 | 3.60 | 4.10 | 3.80 | 3.56 | 4.08 | 3.49 | 3.55 |
| Boltzmann Distribution | 0.000201 | 0.000005 | 0.000001 | 0.022105 | 2.488934 | 0.051326 | 61.73291 | 0.00016 | 30.230442 | 2.92561 | 2.409539 | 0.000006 | 0.043893 | 0.000209 | 0.026297 | 0.011265 | 0.000052 | 0.023915 | 0.031946 | 0.000654 | 0.000532 |  |

**Table S2.** Calculated ^13^C and ^1^H NMR chemical shifts for each conformer of compound
**1-II** and their Boltzmann distribution.

| **Atom** | **Conf. 1** | **Conf. 2** | **Conf. 3** | **Conf. 4** | **Conf. 5** | **Conf. 6** | **Averaged** |
| --- | --- | --- | --- | --- | --- | --- | --- |
| C2 | 141.5 | 141.1 | 141.6 | 141.6 | 141.4 | 142.1 | 141.3 |
| C3 | 111.6 | 110.9 | 112.0 | 111.8 | 111.2 | 112.2 | 111.2 |
| C3a | 122.8 | 123.0 | 123.2 | 122.8 | 123.0 | 123.8 | 123.0 |
| C4 | 124.5 | 124.5 | 124.3 | 124.3 | 124.2 | 125.4 | 124.3 |
| C5 | 121.7 | 121.6 | 121.9 | 121.9 | 121.0 | 123.6 | 121.1 |
| C6 | 130.0 | 130.0 | 130.1 | 130.0 | 129.3 | 131.1 | 129.4 |
| C7 | 115.8 | 115.0 | 115.6 | 115.4 | 114.7 | 115.6 | 114.8 |
| C7a | 135.1 | 134.7 | 135.0 | 134.5 | 134.2 | 135.8 | 134.4 |
| C8 | 171.5 | 171.8 | 171.5 | 171.9 | 172.7 | 170.7 | 172.5 |
| C9 | 159.5 | 158.4 | 160.6 | 165.4 | 159.6 | 163.0 | 159.4 |
| C11 | 37.9 | 35.6 | 38.5 | 40.0 | 37.2 | 39.9 | 36.9 |
| C12 | 64.2 | 62.6 | 57.7 | 56.5 | 57.3 | 66.0 | 58.4 |
| H2 | 8.83 | 8.78 | 8.70 | 8.69 | 8.85 | 8.74 | 8.84 |
| H4 | 8.41 | 8.58 | 8.43 | 8.40 | 8.35 | 8.52 | 8.40 |
| H5 | 7.68 | 7.63 | 7.65 | 7.71 | 7.63 | 7.82 | 7.63 |
| H6 | 7.94 | 7.96 | 7.94 | 8.03 | 8.01 | 7.96 | 8.00 |
| H7 | 7.61 | 7.62 | 7.63 | 7.62 | 7.50 | 7.68 | 7.53 |
| H11 | 3.68 | 3.81 | 3.74 | 3.38 | 3.68 | 3.91 | 3.71 |
| H12 | 3.83 | 3.54 | 3.92 | 4.25 | 3.57 | 3.99 | 3.57 |
| Boltzmann Distribution | 0.027546 | 20.70202 | 0.921613 | 0.039552 | 78.304815 | 0.004454 |  |

© 2015 by the authors; licensee MDPI, Basel, Switzerland. This article is an open access article distributed under the terms and conditions of the Creative Commons Attribution license (http://creativecommons.org/licenses/by/4.0/).
